# Primary adenoid cystic carcinoma of the lung: Clinicopathological features, treatment and results

**DOI:** 10.3892/ol.2015.2859

**Published:** 2015-01-08

**Authors:** MING-MING HU, YING HU, JIA-BEI HE, BAO-LAN LI

**Affiliations:** Department of General Medicine, Beijing Tuberculosis and Thoracic Tumor Research Institute/Beijing Chest Hospital, Capital Medical University, Beijing 101149, P.R. China

**Keywords:** adenoid cystic carcinoma, lung, clinicopathology, immunohistochemistry, treatment, survival

## Abstract

Adenoid cystic carcinoma of the lung (ACCL) is a rare salivary gland-type malignant neoplasm that occurs infrequently as a primary tumor of the airway. Owing to its low incidence, the clinicopathological features, immunohistochemical expression spectrum, treatment and long-term survival have not been fully elucidated. The present study retrospectively assessed the clinical features, immunohistochemical characters, treatment strategy and long-term survival of 34 patients diagnosed with ACCL at the Beijing Chest Hospital, Capital Medical University (Beijing, China) between January 1993 and June 2014. ACCL tended to occur in younger patients, with an approximate male/female ratio of 1:1. The majority of ACCL arose from the central airway. Positive immunochemical staining was found in wide-spectrum keratin (n=17), cytokeratin (CK)7 (n=11), p63 (11/12), S-100 (7/8), vimentin (10/12) and smooth muscle actin (6/9). No staining of thyroid transcription factor-1 (0/14), synaptophysin (0/7), cluster of differentiation 56 (0/7), CK20 (0/4) or chromogranin A (0/4) was observed. In the operable group (n=26), the addition of adjuvant radiotherapy to a positive margin resection (R1 resection) obtained long-term survival times equivalent to that found in patients with a negative margin resection (R0 resection). No significant survival benefit from post-operative radiotherapy was observed in the R0 resection group. For advanced cases, palliative radiotherapy and chemotherapy did not work efficiently. In addition, epidermal growth factor receptor mutation was a rare event in the ACCL patients. The results indicated that surgical resection is the optimal management for ACCL whenever feasible. Adjuvant radiotherapy with R1 resection is able to obtain long-term survival times comparable with those found using an R0 resection. The recommendation of post-operative radiotherapy for all patients with ACCL undergoing resection appears controversial. Owing to a poor response to radiotherapy and chemotherapy, more focus should be placed on the study of advanced ACCL in order to improve overall survival.

## Introduction

Adenoid cystic carcinoma (ACC) is a malignant neoplasm that frequently originates from the salivary glands of the head and neck, but can also take place in the breast, skin, upper digestive tract and lungs (1,2). Formerly, it was referred to as a benign glandular neoplasm, however, ACC is now regarded as a low-grade bronchial carcinoma (3). Primary ACC of the lung (ACCL) arising from the bronchial glands is a particularly rare disease and accounts for only 0.04–0.2% of all primary lung tumors (4,5). Although salivary gland-type tumors generally have an indolent nature, ACCL has a notable tendency for local recurrence and late hematogenous metastases. Due to its rarity, primary ACCL has mainly been reported in small series or case studies. Thus, its precisely clinical and pathological features, clinical course, therapeutic strategy and survival data have not been fully elucidated. The current study summarized 22 years of experience dealing with ACCL in a single institution during the period between January 1993 and June 2014.

## Materials and methods

### Patients

In total, 36 patients who were admitted to the Beijing Chest Hospital, Capital Medical University (Beijing, China) and diagnosed with ACCL between January 1993 and June 2014 were analyzed retrospectively for the present study. Two cases were excluded from the series: One case presented with a sublingual neoplasm concomitantly, except the main bronchus lesion was excluded, and thus it was difficult to identify the primary lesion at that time; and the other case underwent a submandibular tumor resection four years prior to the present study. Therefore, 34 patients entered the final analysis. The medical records were reviewed in order to extract data on demographic characteristics, clinical and pathological features, tumor stage (American Joint Committee on Cancer criteria), treatment and survival (6). The 34 patients were divided into operable (n=26) and non-operable (n=8) groups. All cases underwent bronchoscopy-guided biopsy, which was further corroborated histologically in the surgical specimens. Immunohistochemical staining was conducted in 17 cases to verify the diagnosis of ACCL. The level of carcinoembryonic antigen (CEA) was determined by chemiluminescence, and levels >5 ng/ml were defined as abnormal. This study was approved by the Ethical committee of the Beijing Chest Hospital, Capital Medical University.

### Statistical analysis

Statistical analysis was performed using the SPSS 16 software (SPSS, Inc., Chicago, IL, USA). All categorical variables are presented as counts and percentages. Continuous variables are presented as the median and range. Overall survival time was defined as the interval between primary surgery and mortality due to lung cancer or the date of the last follow-up. The disease-free survival time was defined as the interval between the date of resection and the date of proven detection of recurrence or metastases. Survival curves were generated using the Kaplan-Meier method and the difference in survival was examined using the log-rank test. P<0.05 was considered to indicate a statistically significant difference.

## Results

### Clinicopathological features

Patient characteristics are summarized in Table I. The study consisted of 34 patients with primary ACCL, including 16 males and 18 females, with a median age of 46 years (range, 22–73 years). With respect to smoking information, nearly one-third (32.4%) of patients were current or former smokers, while 67.6% had never smoked. A total of 21 (61.8%) primary lesions were located in the trachea, main bronchus or truncus intermedius, 11 (32.3%) tumors were below the lobar bronchus and 2 (5.9%) tumors were located in the pulmonary parenchyma. Of the 22 patients whose CEA levels were recorded, one patient exhibited an abnormal level. A total of 30 patients were symptomatic when they sought treatment; the most frequent symptom was coughing (n=26; 76.5%), followed by hemoptysis (n=13; 38.2%), shortness of breath (n=10; 29.4%), stridor (n=5; 14.7%), fever (n=3; 8.8%) and chest pain (n=2; 5.9%). The majority of these patients had two or more symptoms. Several patients were incorrectly diagnosed with bronchitis or asthma prior to their visit to the Beijing Chest Hospital, Capital Medical University. Four patients without subjective symptoms visited their doctors for a shadow that was incidentally detected on X-ray or computed tomography (CT) scans of the chest. The median duration between the appearance of the symptoms and the time of diagnosis was 4 months (2 weeks to 42 months). In the operable group, the greatest diameter of the lesions ranged between 1.0 and 7.0 cm, with a median of 2.5 cm. Involvement of the lymph nodes were observed in five patients (N1=3; N2=2). Three of the 26 samples showed infringement of the whole bronchus wall. A total of 16 patients achieved an R0 resection, which meant that the surgical margin was histologically free of disease at the final pathological examination (Table I). Of the 10 patients with microscopically residual tumor at the transected ends, nine patients received post-operative radiotherapy (50–70 Gy, 5–7 weeks) and one succumbed perioperatively.

### Immunohistochemical features

Immunohistochemical examination was conducted in 17 cases. Antibodies to wide-spectrum keratin, actin, vimentin (Vim), S-100 protein, CEA, glial fibrillary acidic protein (GFAP), smooth muscle actin (SMA), cluster of differentiation 56 (CD56), thyroid transcription factor-1 (TTF-1), p63, chromogranin A (CgA), synaptophysin (Syn), cytokeratin (CK)20), CK7, leukocyte common antigen (LCA), caudal-type homeobox 2 (CDX-2), neuron-specific enolase (NSE) and CK5/6 were chosen in different combinational panels to confirm the diagnosis of primary ACCL according to the respective condition of the patient. The positive staining counts of this antigen spectrum was present as follows: Wide-spectrum keratin in 17/17 patients; p63 in 11/12 patients; SMA in 6/9 patients; S-100 in 7/8 patients; Vim in 10/12 patients; CK7 in 11/11 patients; GFAP in 1/3 patients; CEA in 2/9 patients; actin in 2/2 patients; CK5/6 in 1/1 patient; and NSE in 1/1 patient. One sample was positively-stained with the periodic acid-Schiff stain (PAS) method (n=1). Staining was absent for Syn in seven patients, CD56 in seven patients, CK20 in four patients, CgA in four patients, CDX-2 in one patient, TTF-1 in 14 patients and LCA in one patient.

### Treatment of ACC

In the operable group (n=26), all patients selectively underwent plain CT, contrast-enhanced CT, radioisotope bone scans, ultrasound scanning, brain magnetic resonance imaging, blood examinations and lung functional examinations prior to surgery to determine clinical stage and evaluate the feasibility of the procedure. The surgical procedures included tracheal resection (n=4; 15.4%), carinal resection and reconstruction (n=3; 11.5%), (bi)lobectomy (n=9; 34.6%), sleeve lobectomy (n=5; 19.2%) and pneumonectomy (n=5; 19.2%). One patient succumbed to a cardiac arrest within 30 days of a carinal resection and reconstruction. A total of 13 cases received adjuvant radiotherapy (40–70 Gy, 4–7 weeks), while nine cases received neither radiotherapy nor chemotherapy. In the cases with recurrence or metastasis, local or systemic treatment was administered according to the respective condition of the patient, including palliative radiotherapy, systemic chemotherapy, cryotherapy and Chinese herbal medicine. Nine of the 10 patients with positive margins received adjuvant radiotherapy (50–70 Gy, 5–7 weeks). Local recurrence/metastasis occurred during the follow-up of the nine patients; of these patients, cases 3, 5, 8 and 9 received the initial chemotherapy in the Beijing Chest Hospital, Capital Medical University (Table II). Case 3 was administered paclitaxel and cis-platinum (paclitaxel, 175 mg/m^2^, day 1; cis-platinum, 75 mg/m^2^, days 1–3), but the metastatic lesions in each lung progressed subsequent to the first cycle. One cycle of gemcitabine (1,250 mg/m^2^, days 1 and 8) and cis-platinum (75 mg/m^2^, days 1–3) was administered to case 5, which was followed by continuous tumor enlargement in the lung and pleura. Single-drug chemotherapy with pemetrexed (500 mg/m^2^) was attempted as second-line treatment, but also failed. Case 8 was administered one cycle of navelbine (25 mg/m^2^, days 1 and 8), with cis-platinum (75 mg/m^2^, days 1–3) as the first-line treatment and one cycle of paclitaxel (175 mg/m^2^, day 1) and cis-platinum (75 mg/m^2^, day 1) as second-line treatment. Although no response was observed throughout the chemotherapy regimen, the patient remains alive at the time of writing this study. Case 9 received two cycles of paclitaxel (175 mg/m^2^, day 1) and cis-platinum (75 mg/m^2^, days 1–3) treatment, and the lesion was markedly reduced in size. However, six months later, new metastasis appeared. The use of other chemotherapeutic agents (gemcitabine and carboplatin) was attempted in the patient’s district hospital.

In the non-operable group (n=8), three patients were diagnosed with stage IV disease, with multiple lung metastases, and five patients were diagnosed with stage IIIB disease due to the advanced local situation. Palliative treatments, including radiotherapy, systemic chemotherapy, tracheal stent implantation, interventional therapy, high-frequency electrocautery through bronchoscopy and Chinese herbal medicine, were administered to these patients at different time-points in the clinical course. Two stage III patients with lesional invasion into the pulmonary artery were administered primary radiotherapy (the schedued dose was 40 Gy) in the Beijing Chest Hospital, Capital Medical University. The lesions remained unchanged following radiotherapy, so the possibility of surgery was not achieved. One stage III patient was firstly administered a tracheal stent implantation to alleviate airway stenosis; this patient was the only patient alive in the non-operable group after 44 months. One stage III patient was forced to give up subsequent radiotherapy due to aggravation of the symptom of stridor following the initial 2 Gy fraction. Another stage III patient received palliative radiotherapy in a local hospital following the final diagnosis in the Beijing Chest Hospital, Capital Medical University, but the effect was poor. The patient succumbed to respiratory failure one year later. Two stage IV patients received primary chemotherapy in the Beijing Chest Hospital, Capital Medical University; one was administered with gemcitabine (1,250 mg/m^2^, days 1 and 8) and cis-platinum (75 mg/m^2^, day 1), but developed PD due to enlargement of the primary lesion and mediastinal lymph nodes subsequent to two cycles. The other case was treated with navelbine (25 mg/m^2^, days 1 and 8) and cis-platinum (75 mg/m^2^, days 1–3); the subjective symptom was relieved marginally and the lesion remained unchanged. Additionally, one patient insisted on receiving the epidermal growth factor receptor-tyrosine kinase inhibitor (EGFR-TKI), erlotinib, although the patient was aware of the fact that there would probably be no benefit due to a unknown EGFR mutation status. The primary tumor was stable after one month, but the mediastinal lymph nodes became enlarged three months later. It is notable that the EGFR mutation status (exons 18, 19, 20 and 21) from two tumor samples were examined by sequencing method, including a young female who had never smoked, but no mutations were detected.

### Survival

#### Operable group

For the survival analysis, the single case of perioperative mortality was ruled out (n=25). Tumor recurrence/metastasis was identified in nine patients, five of who exhibited residual tumor at the resection margin at the time of the primary surgery (Table II). The local recurrence/metastasis rate was 55.6% (5/9) in the group with positive resection margins, while in the group with negative resection margins, the rate was 25.0% (4/16). The three-, five- and 10-year disease-free survival rates of the operable group were 68.8, 64.3 and 38.5%, respectively. Five mortalities due to ACCL occurred during the follow-up period and these patients survived for 26, 87, 96, 135 and 225 months post-surgery, respectively. For the remaining 20 patients in the operable group, the survival time ranged between two and 253 months. The three-, five- and 10-year overall survival rates of the operable group were 92.9, 91.7 and 70.0% (Table III).

**Figure 1:**
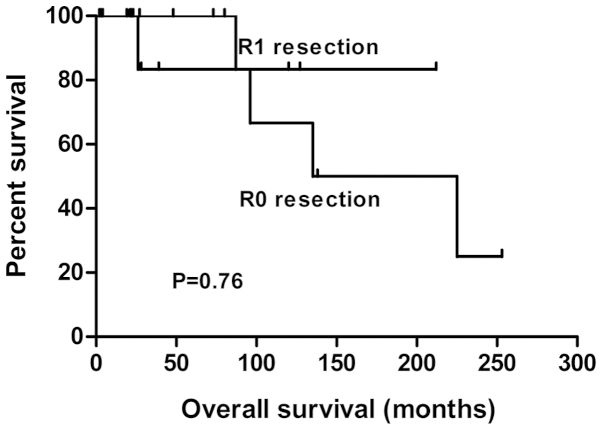
Overall survival curves of patients with primary adenoid cystic carcinoma of the lung who underwent an R0 (n=16) or R1 (n=9) resection.

Although the presence of a positive margin appeared to be associated with recurrence/metastasis, it did not decrease long-term survival. The addition of adjuvant therapy to R1 resection resulted in long-term survival results equivalent to those of an R0 resection. Eight out of the nine patients with positive resection margins who received post-operative radiotherapy were alive at the time of writing this study (Fig. 1; P=0.76). Similarly, the presence of lymph node invasion did not correlate with overall survival time (Table IV). In the subgroup analysis, post-operative radiotherapy did not correlate with local recurrence/metastasis or mortality in the R0 resection group (Table V).

#### Non-operable group

In the non-operable group, eight patients succumbed. Only one patient survived for 44 months post-diagnosis; stent implantation was administered to this patient to alleviate local stenosis. The overall survival period of the patients who succumbed ranged between 12 and 48 months, with a median of 23 months. The three- and five-year overall survival rates for the non-operable group were 50.0 and 0.0%, respectively (Table III).

**Figure 2:**
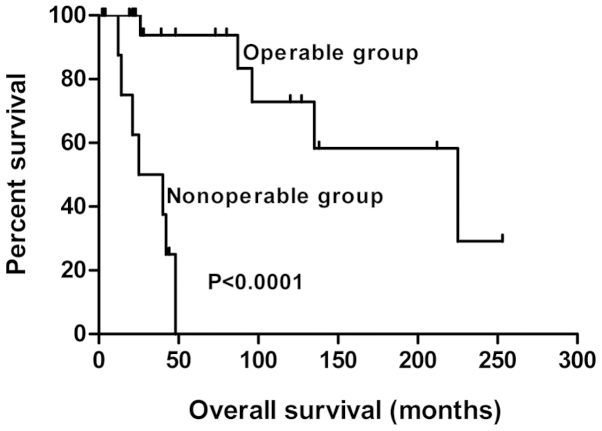
Overall survival curves patients with primary adenoid cystic carcinoma of the lung in the operable (n=25) and non-operable (n=8) groups.

The overall survival curves of the operable and non-operable groups are shown in Fig. 2.

## Discussion

Primary ACCL is a rare salivary gland-type malignant neoplasm that is distributed along the submucosa of the major airways, therefore, a prolonged period is required in order to accumulate a number of cases. In the present study, the clinical features, histopathology and immunohistochemical characters, treatment strategy and follow-up data of 34 patients treated at the Beijing Chest Hospital, Capital Medical University, between 1993 and 2014, was investigated in order to enhance our understanding of primary ACCL.

Compared with other primary lung cancers, ACCL is quite different in terms of its demographic characteristics. ACCL tends to occur in younger patients, with an approximate male/female ratio of 1:1 (7,8). However, there is a marked predominance of males in the incidence of other lung cancers (8,9). All smoking information was collected in the present study, and the majority of the cohort (67.6%) were found to be never smokers. This was generally accordant with other studies (7,10). Therefore, smoking does not appear to be associated with the etiology of ACCL. In the present series, coughing was the most common symptom of ACCL, followed by hemoptysis and shortness of breath. Several patients were previously wrongly treated for asthma and bronchitis. Occasionally, patients were asymptomatic, until detected in imaging examination, particularly in those patients with peripheral ACCL (11).

ACCL presents with particular histopathological features; in the present study, lymphatic metastases were relatively uncommon, occurring in only five patients undergoing resection. However, due to the extensive spread along the major axis of the trachea at the time of diagnosis, residual tumors at the resection margin are not rare. However, resection with upper and lower margins of 1 cm from the macroscopic tumor, cannot guarantee an R0 resection. In such cases, post-operative radiotherapy is indispensable (12). In total, >90% of ACCLs arise in the central airway, thus accounting for the small number of reported cases in the peripheral lung (13). ACC of the peripheral lung must therefore be distinguished from metastatic lung tumors (13,14). In the present study, two cases of peripheral ACCL, without bronchoscopic evidence, were confirmed. These patients did not exhibit salivary gland neoplasms five years prior to or following surgery, and the diagnosis was supported by immunohistochemistry in the post-operative tumor tissues.

Although hematoxylin-eosin staining remains the principal method used for the diagnosis of ACCL, it can occasionally be misdiagnosed as carcinoid or mucinous adenocarcinoma. In these cases, immunohistochemistry is a useful tool to enhance the accuracy of histological diagnosis (15). To the best of our knowledge, the number of studies investigating the immunohistochemistry characteristics of ACCL is sparse. In the present study, immunostaining of wide-spectrum keratin (17/17), CK7 (11/11) and actin (2/2) was positive in all samples examined for these markers, and the vast majority showed positivity for p63 (11/12), S-100 (7/8), Vim (10/12) and SMA (6/9). These immunohistochemical findings were comparable to the study by Moran *et al* (16). No staining for TTF-1 (0/14), Syn (0/7), CD56 (0/7), CK20 (0/4) or CgA (0/4) was observed. Only partial staining was present for CEA (2/9) and GFAP (1/3) antibodies. The expression of TTF-1, a transcription factor specifically expressed in the thyroid gland and lungs, is found in 60–70% of pulmonary adenocarcinomas. An *et al* (17) reported 12 cases of primary ACCL without TTF-1 staining, which was comparable with our result, but a case of peripheral ACCL with positive TTF-1 has been reported (14).

The optimal management for ACCL is surgical resection whenever feasible. in the present study, 26 patients comprised the operable group. The three-, five- and 10-year overall survival rates were 92.9, 91.7 and 70.0%, respectively. Patients with positive margins were all administered adjuvant radiotherapy (n=9). No difference was found in overall survival time between those who underwent R0 and R1 resections (Fig. 1). The longest survival time in the operable group was 253 months, without recurrence or metastasis. The combination treatment of surgery and radiotherapy in the patients who underwent R1 resection resulted in long-term survival that was comparable that of patient who underwent R0 resection, although positive cases were prone to recur locally. Eight patients with positive resection margins were alive at the time of writing this study, and three patients survived for 120, 127 and 212 months, respectively, post-surgery. It is our belief that surgery should be the first therapeutic option, but that the security of the patient and the healing of the anastomosis is more important than more tumor-free margins (18–20). A survival analysis was also performed in the patients who underwent an R0 resection (n=16) in the present study. In four cases receiving radiotherapy, one patient experienced recurrence/metastasis and succumbed at 87 months post-surgery. In the 12 cases without radiotherapy, three patients experienced recurrence/metastasis and succumbed 96, 135 and 225 months post-surgery, respectively. In the R0 resection group, adjuvant radiotherapy was not found to correlate with overall survival. The possible benefit of post-operative radiotherapy on overall survival in R1 resection only was not analyzed, as all patients were administered radiotherapy for prudential reasons. Based on our observations, it appeared to be controversial to recommend post-operative radiotherapy for all patients with ACCL undergoing resection. To date, concrete data on the benefit of adjuvant radiotherapy in R0 resection has not been reported, but the conclusions of the present study also agreed with another larger sample study (n=64) (19). Similarly, the presence of positive lymph nodes did not seem to decrease survival times, as reported by Regnard *et al* (19) and Maziak *et al* (20). However, as ACCL is likely to grow along the major axis of the trachea and more extensive invasion than is found in the pre-operative evaluation is often found intraoperation, sophisticated skills are required for surgical treatment.

With regard to advanced ACCL, palliative chemotherapy has seldom been described in the literature. One case sensitive to uracil-tegafur and cisplatin plus radiotherapy has been reported (21). In the present study, in the five cases receiving palliative chemotherapy, only one showed sensitivity to paclitaxel and cis-platinum treatment. Four patients benefited marginally from platinum-based double chemotherapy, which is the most frequently chosen first-line therapy for advanced non-small cell lung cancer (NSCLC). In addition, four cases in the non-operable group responded poorly to palliative irradiation. EGFR-TKIs are recommended as the first-line targeted therapy in cases of advanced NSCLC with an activating EGFR mutation. EGFR kinase domain mutations are rare in salivary gland carcinomas (22,23). In the present retrospective study, two cases provided samples for examination, including a young, non-smoking female, who was regarded as more prone to an EGFR mutation. Another non-smoking female unaware of the EGFR mutational status insisted in being administered erlotinib and exhibited a stable lesion for one month, prior to progression of the local lymph nodes after three months. Taking these data into consideration, a novel therapeutic strategy must be sought for advanced ACCL. The use of imatinib has been reported for one advanced case and achieved a satisfactory effect (24).

In conclusion, the present study has described the demographic characteristic, clinicopathological and immunohistochemical features, clinical treatment and long-term survival of 34 patients with ACCL. As a retrospective study, the collection and review of these data clearly has limitations. Additionally, the study lacked statistical power due to the relatively small number of patients, although the cases were accumulated over a 22-year time span. Furthermore, three different subtypes of this tumor, tubular, cribriform and solid, have been described by pathologists, with the solid subtype assumed to be associated with a more aggressive phenotype (16). Another shortcoming of the present study was that subtype identification was not performed.

## Figures and Tables

**Table I:** Clinicopathological characteristics of patients (n=34)with primary adenoid cystic carcinoma of the lung.

Variables	Value
Median age (range), years	46 (22–73)
Gender, n (%)	
Male	16 (47.1)
Female	18 (52.9)
Smoking status, n (%)	
Current or ever	11 (32.4)
Never	23 (67.6)
CEA level (n=22), n (%)	
≥5 ng/ml	1 (4.5)
<5 ng/ml	21 (95.5)
Tumor location, n (%)	
Trachea, main bronchus	17 (50.0)
Truncus intermedius	4 (11.8)
Lobar bronchus	8 (23.5)
Segmental bronchus	3 (8.8)
Periphery	2 (5.9)
Operable group (n=26)	
Node invasion, n (%)	
N0	21 (80.8)
N1	3 (11.5)
N2	2 (7.7)
Resection margin, n (%)	
Negative	16 (61.5)
Positive	10 (38.5)
Pathological stage, n (%)	
I	11 (42.3)
II	6 (23.1)
IIIA	7 (26.9)
IIIB	2 (7.7)
Median tumor size (range), cm	2.5 (1.0–7.0)
Surgical procedure, n (%)	
Tracheal resection	4 (15.4)
Carinal resection and reconstruction	3 (11.5)
(Bi)lobectomy	9 (34.6)
Sleeve lobectomy	5 (19.2)
Pneumonectomy	5 (19.2)
Adjuvant therapy (n=25), n (%)	
No	9 (36.0)
Chemotherapy and radiotherapy	6 (24.0)
Chemotherapy only	3 (12.0)
Radiotherapy only	7 (28.0)
Non-operable group (n=8)	
Clinical stage, n (%)	
III	5 (62.5)
IV	3 (37.5)

CEA, carcinoembryonic antigen.

**Table II:** Summarized information on tumor recurrence/metastasis and mortality in the operable group.

Case no.	Recurrence/metastasis (site)	Resection margin	Status	DFS, months	Follow-up, months
1	Bronchial resection margin	Positive	Alive	3	120
2	Brain, lung, bone	Negative	Succumbed	83	96
3	Kidney, lung, bone, bronchial resection margin	Negative	Succumbed	197	225
4	Lung, liver	Negative	Succumbed	128	135
5	Lung, pleura	Positive	Alive	7	21
6	Bronchial resection margin	Positive	Alive	13	28
7	Lung, pleura	Positive	Succumbed	16	26
8	Bronchial resection margin	Positive	Alive	2	127
9	Lung, mediastinal lymph nodes, liver	Negative	Succumbed	78	87

DFS, disease-free survival.

**Table III:** Survival information for the operable (n=26) and non-operable (n=9) groups.

	Overall survival rate, %			Disease-free survival rate, %		
		
Group	3-year	5-year	10-year	3-year	5-year	10-year
Operable	92.9	91.7	70.0	68.8	64.3	38.5
Non-operable	50.0	0.0	0.0	-	-	-

**Table IV:** Effect of lymph node involvement on the survival time of patients with primary adenoid cystic carcinoma of the lung in the operable group.

Group	Patients, n	Recurrence/metastasis, n	Mortality, n	Follow-up, months
Positive	4	2	1	21, 22, 39, 87^a^
Negative	21	7	4	48 (2–253)^b^ 26^a^, 96^a^, 135^a^, 225^a^

a Patient had succumbed.

b Data is presented as median (range).

**Table V:** Effect of adjuvant radiotherapy on the survival of patients with primary adenoid cystic carcinoma of the lung who underwent an R0 resection.

Group	Patients, n	Recurrence/metastasis, n	Mortality, n	Follow-up, months
Radiotherapy	4	1	1	2, 19, 87^a^, 253
Without radiotherapy	12	3	3	4, 22, 22, 23, 27, 48, 73, 80, 96^a^, 135^a^,138, 225^a^

a Patient had succumbed.
